# The impact of early death on birth counts in the United States, 1950 to 2019

**DOI:** 10.1093/pnasnexus/pgae058

**Published:** 2024-06-07

**Authors:** Antonino Polizzi, Andrea M Tilstra

**Affiliations:** Department of Sociology, University of Oxford, 42-43 Park End Street, Oxford OX1 1JD, UK; Leverhulme Centre for Demographic Science, University of Oxford, 42-43 Park End Street, Oxford OX1 1JD, UK; Nuffield College, University of Oxford, New Road, Oxford OX1 1NF, UK; Nuffield Department of Population Health, University of Oxford, Richard Doll Building, Old Road Campus, Oxford OX3 7LF, UK; Department of Sociology, University of Oxford, 42-43 Park End Street, Oxford OX1 1JD, UK; Leverhulme Centre for Demographic Science, University of Oxford, 42-43 Park End Street, Oxford OX1 1JD, UK; Nuffield College, University of Oxford, New Road, Oxford OX1 1NF, UK; Nuffield Department of Population Health, University of Oxford, Richard Doll Building, Old Road Campus, Oxford OX3 7LF, UK

**Keywords:** United States, demography, mortality, fertility, counterfactual

## Abstract

In a previous issue of *PNAS Nexus*, Bor et al. quantified the number of “missing Americans”—the deaths that would have been averted if the United States had experienced the mortality conditions of other wealthy nations. In 2019 alone, their estimates indicate that more than 100,000 individuals in reproductive ages (15–49 years) would have survived. The concept of the “missing Americans” is a valuable one, but here we argue that it is incomplete because it does not include children that would have been born to those who died an early death. We examine 3 indicators to assess the strength of the mortality–fertility nexus at the population level, showing that mortality more negatively affects birth counts in the United States than in other wealthy nations. Using the mortality conditions in other wealthy nations as a reference, we estimate that between 2010 and 2019 alone, approximately 200,000 children were not born in the United States due to the premature death of their potential mothers. Our findings highlight that improving morbidity and mortality among people of reproductive age—without compromising their reproductive autonomy—is critical in the United States.

**Significance Statement**

Higher mortality in the United States compared with other wealthy nations affects (i) the number of individuals still alive at any point in time and, indirectly, (ii) the number of individuals born into the population. The concept of the “missing Americans,” as defined by Bor et al., captures the former effects of the mortality divergence between the United States and its peers—but not the latter. We argue that the concept of the “missing Americans” should be broadened to include never-born children due to early mortality among potential parents and that contemporary demography could benefit from studying the mortality–fertility nexus, even in populations in which mortality rates appear low.

## Introduction

In a previous issue of *PNAS Nexus*, Bor et al. provided an estimate of the number of deaths that would have been averted if the United States had experienced the mortality conditions of other wealthy nations (1). In 2019 alone, their estimates point to a total of 600,000 Americans that “went missing,” or individuals that died an early death.

The concept of the “missing Americans” is a valuable tool to illustrate the US mortality disadvantage because it quantifies the comparatively high levels of mortality in the United States using an intuitive metric. It also complements the often difficult-to-communicate summary indicators of all-cause mortality—such as life expectancy at birth or age-standardized mortality rates—that demographers use when sharing their findings with a broader audience. Finally, “missing Americans” points to the avoidable consequences of early mortality for society more broadly. As Bor et al. argue, preventable mortality and underlying health issues may impact individuals’ ability to contribute to the economic, political, and social spheres and may negatively affect the socioeconomic and emotional well-being of family members and members of the social network of sick or deceased individuals (1).

The enumeration of missing populations has a long tradition in demography, as evidenced by the abundance of literature on “missing girls,” “missing women,” or “missing females” (2).^1^ “Missingness” here is not defined as the lack of knowledge of the whereabouts of a person due to their disappearance by their own will or by the force of another person. Rather, it refers to the fact that a given society would include more individuals if demographic conditions were or had been different. For example, Bongaarts and Guilmoto estimated that 126 million more girls and women would have been alive worldwide in 2010 if age-specific sex ratios were not biased toward males (5). Skewed population sex ratios are typically explained by patriarchal structures that encourage sex-selective abortion, infanticide, and childcare practices, as well as differential access to resources, resulting in biased sex ratios at birth and elevated female mortality (6). Thus, the literature on missing females calls attention to the fact that social inequality frequently manifests itself in fertility and mortality outcomes, and the concept of the “missing Americans” follows this tradition.

Despite drawing attention to some important sequelae of early mortality at the individual and societal levels, “missing Americans,” as currently defined by Bor et al., does not account for the forgone fertility due to mortality before the end of the reproductive period. Of the 600,000 total excess deaths observed in 2019, more than 100,000 were in reproductive ages (15–49 years). Many of these individuals would have had children if they had not died prematurely, leading to “missing births.”^2^ We argue that expanding the concept of “missing Americans” to account for the nexus between mortality and fertility provides a more comprehensive assessment of the negative consequences of the US mortality environment for society.

Thinking about the fertility implications of early mortality is not a new approach in demographic research. Nearly 30 years ago, demographers asked questions like “How many Americans are alive because of twentieth-century improvements in mortality?” (7) or “How many Americans might have been alive in the twentieth century?” (8) and used a counterfactual population projection approach to estimate the number of Americans that “literally owe their lives to health progress” (7) or that “could be alive … had mortality in the United States … been the lowest possible at the time” (8). Estimates from these studies already included lives saved directly in the first generation and those saved indirectly in subsequent generations.

Besides projection methods, formal demographic summary indicators of the mortality–fertility nexus, such as the net reproduction rate, have existed for decades (9). These easy-to-calculate indicators are regularly reported for all countries by the United Nations Department of Economic and Social Affairs (10). In high-income contexts, there is a tendency to think that mortality before the end of the reproductive period is sufficiently low to assess mortality and fertility conditions separately using period life expectancy at birth and the total fertility rate. However, considering the rising mortality gap between the United States and its peer countries that has resulted in excess premature mortality among people of reproductive ages, we argue for the continued and extended use of mortality–fertility indicators for high-income countries, especially in a comparative setting.

In this article, we review 3 existing summary indicators that can be used to communicate the magnitude of the mortality–fertility nexus in the United States, both within the community of demographic researchers and to a broader audience: (i) the probability of survival from birth to age 50 years; (ii) reproductive-age life expectancy (RALE); and (iii) the reproduction–survival ratio (RSR). Moreover, we advocate for the continued utility of counterfactual population projections to study the nexus between mortality and fertility at the population level. We review the principles behind counterfactual population projections, illustrating how this method can be used to study the population impact of short-term demographic shocks or longer-term demographic disadvantages. Our empirical application shows that the contemporary United States performs worse on the 3 mortality–fertility indicators than its peer nations, indicating that mortality more negatively affects birth counts in the United States. Using the mortality conditions in other wealthy nations as a reference, our counterfactual population projections suggest that between 2010 and 2019 alone, approximately 200,000 children were not born in the United States due to the premature death of their potential mothers.

Beyond measurement, we emphasize that improving the morbidity and mortality of reproductive-aged people is critical in the United States. Pronatalist and nationalist ideologies often aim to restrict women's reproductive autonomy to increase future population size, usually at the expense of the health of pregnant and birthing people currently alive (11). Building on existing frameworks (12), the indicators and methods examined in this article provide tools to quantify the extent to which US individuals are constrained in their freedom to start and raise a family on their own terms. The constraint considered here is the risk of death before the end of the reproductive period. We argue that the unconditional focus on the physical integrity and reproductive autonomy of those alive today can also reap benefits tomorrow by enabling individuals to participate actively in society, embedded in kinship and social networks that are not disrupted by premature mortality.

## Quantifying the mortality–fertility nexus

### Probability of survival to age 50 years (ℓ_50_)

The period life table is a demographic tool that allows for the calculation of different summary indicators of survival under the assumption that the mortality conditions observed in a given year and population were held constant for the individuals born in that year (13).^3^ For example, US life expectancy at birth ($e_{0}$) in the year 2010 is not a forecast of the actual length of life of a person born in the United States in that year. Rather, it indicates the average number of years that a newborn could expect to live under the mortality conditions found in the United States in 2010. Thus, the life table can succinctly summarize mortality conditions observed across a wide age range. Unlike other summary indicators of mortality, such as the crude death rate, indicators derived from the life table are not affected by the age structure of the population for which they are calculated, allowing for a straightforward comparison of these indicators across time and across populations.

Assuming a birth cohort (life table radix) of size 1, the $\ell_{x}$ column of the life table indicates the probability of survival from birth to age *x* under the life table assumptions described previously. For example, $\ell_{50}$ is the probability of surviving from birth past the end of the reproductive period, i.e. to age 50 years.^4^

### Reproductive-age life expectancy

Temporary life expectancy, _*n*_*e*_*x*_, is a life table indicator of the average number of years a person that survived to age *x* could expect to live before age *x* + *n* under the current mortality conditions (14). This indicator can reach a maximum value of *n* years, in which case there would be no mortality between ages *x* and *x* + *n*. For example, RALE, or _35_*e*_15_, is the average number of years a 15-year-old could expect to live before the end of the reproductive period (i.e. before age 50 years), with a maximum value of 35 years (15).

### Reproduction–survival ratio

The gross reproduction rate (GRR) is a summary indicator of fertility that follows a similar logic to the life table. It indicates the average number of children of the same sex that an individual of a given sex would bear throughout their lifetime if the fertility conditions observed in a given year and population were held constant (13). GRR assumes no mortality before the end of the reproductive period and is, thus, sometimes regarded as an indicator of “potential reproductivity” (16) in the absence of mortality among potential parents. In contrast, the net reproduction rate (NRR) assumes that individuals were exposed to both the fertility and mortality conditions observed in a given year and population (13). The ratio of NRR to GRR, or the RSR, indicates the share of potential fertility that “survives the effects of mortality” (16) among potential parents. By standardizing for potential fertility in the denominator, RSR can be compared across time and across populations without being biased by differences in the levels of fertility.

Traditionally, GRR, NRR, and RSR focus on the birth of female children to females. Given our interest in the implications of early death for total birth counts, we modify these indicators to account for the birth of children of any sex to females (17).

Table 1 provides an overview of the 3 mortality–fertility indicators, showing the most common interpretation of each indicator, the formula used to calculate each indicator, and how deaths at earlier vs. later ages affect each indicator.

**Table 1.** Demographic indicators of the mortality–fertility nexus.

Indicator	Interpretation^a^	Formula^b^	Maximum value	Deaths accounted for	Impact of deaths at different ages
$\ell_{50}$	Probability of survival to age 50 years	∏490 px1	1	Age <50 years	Equal impact
RALE	Average number of years lived between ages 15–49 years, conditional on survival to age 15 years	∑1549Lx1 ℓ15	35	Ages 15–49 years	Stronger impact of earlier deaths
RSR	Share of potential fertility that survives effects of mortality among potential parents	∑1549Lx1 ×Fx1 ∑1549Fx1	1	Age <50 years	Stronger impact of earlier deaths; equal impact of deaths age <15 years

^a^Under the assumption that current mortality and fertility conditions are held constant and life table radix $\ell_{0}=1$.

^b^_1_ *p*_*x*_ = probability of surviving from age *x* to age *x + 1*; _1_*L_x_* = person-years lived between ages *x* and *x* + *1*; _1_*F_x_* = (two-sex) fertility rate between ages *x* and *x* + *1*.

### Counterfactual population projection

$\ell_{50}$ , RALE, and the RSR are age-standardized summary indicators of mortality and/or fertility conditions in a given year and population. These indicators also assume stability of the underlying demographic conditions. While this means that mortality and fertility conditions can be straightforwardly compared across time and across populations, the real-world implications of different demographic circumstances heavily depend on the age-structure of the individuals experiencing these circumstances. Thus, following previous studies (18, 19), Bor et al. calculated the number of missing Americans by counterfactually subjecting the observed US population in each year to the age-specific mortality rates found in a group of peer countries.

However, the consequences of early mortality for birth counts are not limited to the year of observation. For example, a 30-year-old that died in 1990 would have been able to have children until 2009, if the premature death had been avoided. Thus, quantifying the fertility implications of mortality before the end of the reproductive period requires a counterfactual approach with a longer-term perspective—one in which the missing Americans are assumed not only to survive, but also to have children later on. Research on the population implications of COVID-19 has popularized again an approach known as “counterfactual population projection” (20) to study the long-term effects of demographic shocks (21–23). Here, the population before the shock is projected forward with the cohort component method (13) under 2 scenarios: (i) a baseline scenario, in which the starting population is exposed to the age-specific mortality, fertility, and migration rates actually observed during the shock; and (ii) a counterfactual scenario, in which the starting population is exposed to counterfactual demographic conditions that would have likely prevailed in the absence of the shock. The difference in any indicator between the two scenarios, such as the number of live births, represents the population effect of the demographic shock. This approach can be applied for different time scales to quantify the short- and/or long-term implications of the demographic shock. In addition, we contend that counterfactual population projections are not limited to the study of instantaneous demographic shocks but can also be applied to study the effects of longer-term demographic disadvantages. For example, in the context of the missing Americans, it is possible to study short- and long-term differences in birth outcomes if the United States had experienced the more favorable mortality conditions of its peer countries (8).

While the counterfactual projection of live births takes inspiration from the previously discussed demographic indicators, especially the RSR, it is an independent approach to quantify the implications of early death for birth counts. Compared with the demographic indicators, the strength of the counterfactual projection approach lies in its ability to account for changes in the mortality and fertility conditions across the lifetimes of multiple birth cohorts, even if these cohorts are only partially observed. The projection approach is also able to account for population size, population age structure, and the impact of migration, the latter being an important source of population growth and change (24).

## Materials and methods

We compared the United States with a group of peer countries to illustrate the extent to which US birth counts have been impacted by the unfavorable US mortality environment highlighted in previous studies (25, 26). In line with Bor et al., we varied our group of peer countries to comprise (i) the 5 largest Western European countries—France, Germany, Italy, Spain, and the United Kingdom; (ii) the other G7 (Group of Seven) countries—Canada, France, Germany, Italy, Japan, and the United Kingdom; and (iii) 21 other wealthy nations—Australia, Austria, Belgium, Canada, Denmark, Finland, France, Germany, Iceland, Ireland, Italy, Japan, Luxembourg, The Netherlands, New Zealand, Norway, Portugal, Spain, Sweden, Switzerland, and the United Kingdom. Our analysis was restricted to (early) death in the female population and, where applicable, accounted for the birth of children of any sex.

Using data from the 2022 United Nations World Population Prospects for the period 1950–2100, we constructed annual life tables from estimated and forecasted information on female deaths and female exposures by single year of age and calculated age-specific fertility rates from estimated and forecasted information on births to females and female exposures by single year of age (10). We used the formulas reported in Table 1 and derived values of $\ell_{50}$, RALE, and the RSR for the United States, each of the peer countries, and the population-weighted average of the peer countries for the period 1950–2019.

By comparing annual birth counts in a baseline (with US mortality) and a counterfactual (with peer mortality) projection scenario, we determined the annual number of children that were only (“additional births”) or were not (“missing births”) born because the United States did not experience the mortality conditions of other wealthy nations beginning in 1950. To this end, we applied standard cohort component methodology (13) to estimated and forecasted US population counts reported in the United Nations World Population Prospects. Among the additional births, we distinguished between children born only because their mothers (second generation) or (great-)grandmothers (third and higher generations) survived under US mortality conditions but would have died under peer mortality conditions. Similarly, among the missing births, we distinguished between children not born because their potential mothers (second generation) or (great-)grandmothers (third and higher generations) died under US mortality conditions but would have survived under peer mortality conditions (8). This approach is described in more detail in the Supplementary Information Text. For the main analysis, we commenced and ceased our projections on January 1, 1950, and January 1, 2020, respectively. In supplementary analyses, we extended the projection period to January 1, 2050.

In the main manuscript, we report results for the comparison between the United States and the group of other wealthy nations. Results for the other comparison groups can be found in the Supplementary Information (Figures S1–S8). Replication data and code for all figures are available through our Open Science Framework repository: https://doi.org/10.17605/osf.io/z5djb.

## The US mortality–fertility nexus

**Fig. 1.**
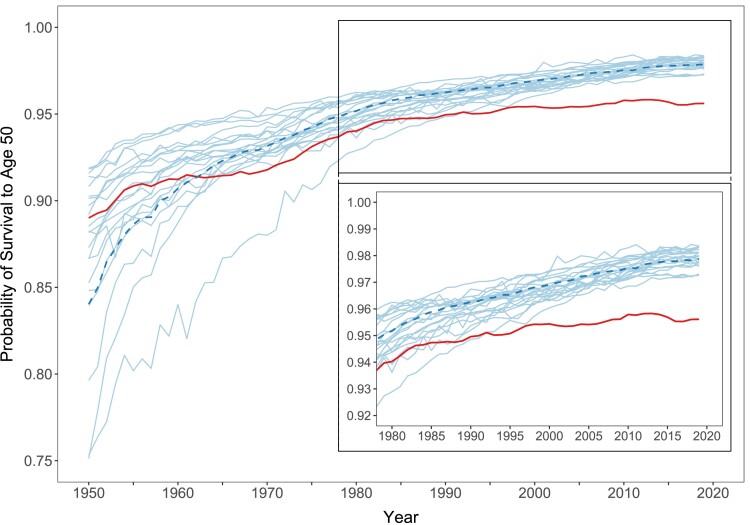
Female probability of survival to age 50 years ($\ell_{50}$) in the United States and 21 other wealthy nations, 1950–2019. *Notes*: Thick solid line = United States; thick dashed line = population-weighted average of the other wealthy nations; thin solid lines = country-specific trends for each of the other wealthy nations. Inset zooms in on period 1980–2019 for better readability. *Source*: Authors’ calculations based on data from United Nations World Population Prospects 2022.

Figure 1 shows trends over the period 1950–2019 in $\ell_{50}$ in the United States and the group of other wealthy nations. The inset in Figure 1 highlights the most recent period, 1980–2019. The thick solid (red) line shows the United States, while the thick dashed (dark blue) line shows the average among the peer countries. Each peer country is represented by a thin solid (light blue) line. Overall, levels of $\ell_{50}$ were high and close to the theoretical maximum of 1. This is especially noticeable at the end of the observation period (2019), where all countries reached values of $\ell_{50}$ above 95%.

In the 1950s, the United States had above-average probabilities of survival to age 50 years. By the mid-1960s, though, the United States stagnated while improvements continued for the peer countries, contributing to the emergence of a US disadvantage in $\ell_{50}$. The gap remained relatively stable during the 1970s but began widening in the 1980s, driven by larger improvements for the peer countries. By the end of the 1990s, $\ell_{50}$ in the United States was lower than in all other wealthy nations. During the 2010s, deteriorations in the United States amid improvements in the peer countries led to further increases in the US $\ell_{50}$ disadvantage. While the United States recovered slightly in the years before the COVID-19 pandemic, the gap remained substantial. If mortality conditions were held at 2019 levels, the probability of survival to age 50 years would be 97.9% in the other wealthy nations but only 95.6% in the United States.

**Fig. 2.**
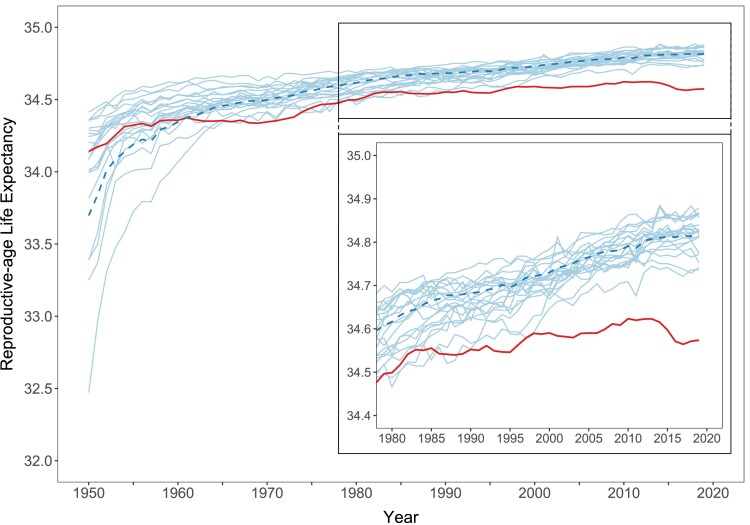
Female RALE in the United States and 21 other wealthy nations, 1950–2019. *Notes*: Thick solid line = United States; thick dashed line = population-weighted average of the other wealthy nations; thin solid lines = country-specific trends for each of the other wealthy nations. Inset zooms in on period 1980–2019 for better readability. *Source*: Authors’ calculations based on data from United Nations World Population Prospects 2022.

Figure 2 shows trends in RALE in the United States and the other wealthy nations between 1950 and 2019. Overall, trends followed a similar pattern as $\ell_{50}$, suggesting that mortality before age 15 was not the underlying driver of the patterns seen in Figure 1. As with $\ell_{50}$, values of RALE in the United States and the other wealthy nations have reached levels close to the theoretical maximum of 35 years, with RALE estimated to surpass 34.5 years in 1981 and 1971 in the United States and the peer average, respectively. In the early 2010s, RALE declined in the United States but recovered somewhat in the late 2010s. If mortality conditions observed in 2019 were held constant, 15-year-olds in the United States would, on average, live 2.9 fewer months before age 50 years than their counterparts in other wealthy nations. While on the individual level, this difference may seem small, Bor and colleagues highlighted the large number of years of life lost in the United States at the population level. This loss, in turn, may have meaningful implications for population-level birth counts, as we illustrate below.

**Fig. 3.**
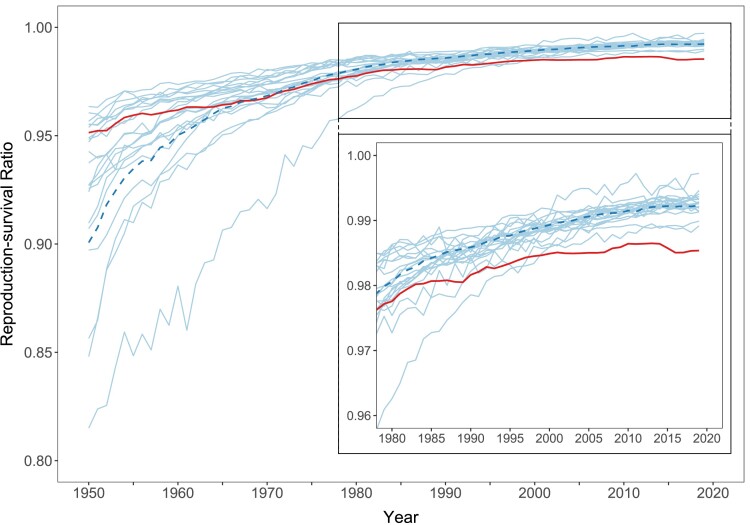
Female RSR in the United States and 21 other wealthy nations, 1950–2019. *Notes*: Thick solid line = United States; thick dashed line = population-weighted average of the other wealthy nations; thin solid lines = country-specific trends for each of the other wealthy nations. RSR accounts for the birth of children of any sex. Inset zooms in on period 1980–2019 for better readability. *Source*: Authors’ calculations based on data from United Nations World Population Prospects 2022.

Echoing our findings for $\ell_{50}$ and RALE in Figures 1 and 2, Figure 3 shows a lower female RSR in the United States compared with the group of other wealthy nations starting in the 1960s. Assuming that (i) mortality and fertility conditions were held at 2019 levels; (ii) deceased women would have had the same fertility as surviving women; and (iii) there was no international migration, the United States would realize 98.5% of its fertility potential, while this level would be somewhat higher, at 99.2%, in the peer countries.

**Fig. 4.**
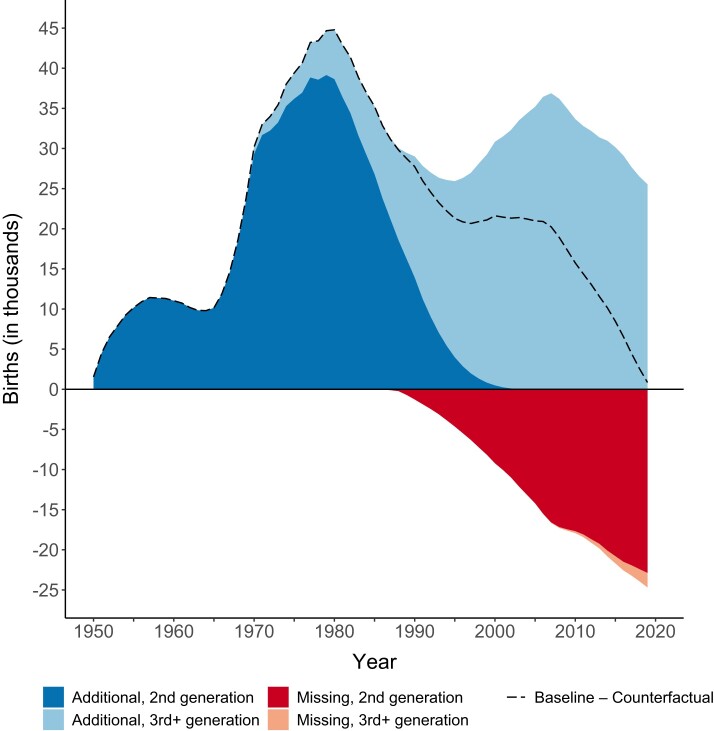
Additional births and missing births in the United States, 1950–2019. *Notes*: Areas = children that were only (pointing upward) or were not (pointing downward) born in the United States each year because the country did not experience the mortality conditions of 21 other wealthy nations beginning in 1950. *Source*: Authors’ calculations based on data from United Nations World Population Prospects 2022.

Finally, Figure 4 shows the annual number of additional and missing births across the period 1950–2019. Areas pointing up (and in blue) indicate US live births that would not have occurred in a counterfactual scenario in which the mortality conditions of other wealthy nations applied beginning in 1950. Conversely, areas pointing down (and in red) indicate live births that would have only occurred under the mortality conditions of other wealthy nations. The dark and light shades disaggregate the additional and missing births further by generation (i.e. second generation vs. third and higher generations), as described in the Materials and methods. The dashed line represents the total annual difference in the number of live births under the baseline (with US mortality) and counterfactual (with peer mortality) population projection scenarios and is equal to the sum of all (blue and red) areas.

Compared with the counterfactual scenario, the United States experienced more live births in each year of the period 1950–2019 (dashed line). Until the 1980s, this was predominantly because of children born only because their mothers’ lives were saved under US mortality conditions, i.e. additional births in the second generation (dark blue area pointing upward). Since then, the importance of children born only because their (great-)grandmothers’ lives were saved under US mortality conditions has increased, i.e. additional births in the third and higher generations (light blue area pointing upward). However, since the mid-1980s, the United States has also seen an increasing number of missing births in the second generation (dark red area pointing downward). Across the decade 2010–2019, around 200,000 children were not born in the United States because their potential mothers died prematurely under US mortality conditions. Of these missing births, about 23,000 would have occurred in 2019 alone. Missing births in the third and higher generations, i.e. children not born because their potential (great-)grandmothers died prematurely under US mortality conditions, played only a minor role during the period 1950–2019 (light red area pointing downward). Using partially observed and forecasted information on population, births, and deaths in the United States and other wealthy nations for the period after 2019, we project that in each year until 2049, the United States will see fewer births than in a counterfactual scenario in which the mortality conditions of other wealthy nations applied beginning in 1950 (see Figure S9). Thus, although the US reproductive-age mortality advantage in the 1950s and 1960s initially resulted in additional births, the persistent disadvantage in reproductive-age mortality that began to emerge in the 1960s is projected to have an offsetting effect on US birth counts in the medium and long terms.

## Discussion

In a previous issue of *PNAS Nexus*, Bor et al. estimated the number of missing Americans—early deaths in the United States due to higher mortality than in other wealthy nations. In this article, we aim first to broaden the concept of “missing Americans” by estimating the number of missing births—the children that would have been born to those who died an early death. Using the mortality conditions in other wealthy nations as a reference, our counterfactual population projections suggest that between 2010 and 2019 alone, approximately 200,000 children were not born in the United States due to the premature death of their potential mothers. The early death of Americans thus has important implications for birth counts.

Our counterfactual projection approach—akin to those used by White and Preston (7) and Muszyńska and Rau (8)—explicitly incorporates information on the population age structure and mimics the movement of actual birth cohorts through time. While estimates provided here mostly show consequences of the past, it is possible to provide projections of forgone fertility due to mortality in the near and more distant future (23). Using partially observed and forecasted information on population, births, and deaths in the United States and other wealthy nations, we project that the United States will continue to experience missing births in the next decades, while the number of additional births will continue to decline.

The effects of mortality on population-level birth counts can compound over time and across generations. Focusing on a 100-year period (1900–2000), Muszyńska and Rau provided estimates of never-born children due to the early death of potential parents, grandparents, and great-grandparents (8). They estimated that more than 700,000 additional children could have been born in the year 2000 alone if the United States had had the highest life expectancy recorded in each year of the 20th century. Counterfactual population projections have been criticized in the past for stretching the projection time window and incorporating the contributions from population momentum (i.e. the fact that never-born children would have had children) (27). While our approach does not fully avoid this issue, we focus on a time period in which the (great-)grandchildren of the missing Americans only make a minor contribution to the total number of missing births, as seen by the light red area pointing downward in Figure 4.

Our second contribution is to compare the United States with other wealthy nations on 3 easy-to-calculate summary indicators of the mortality–fertility nexus: (i) the probability of survival to age 50 years ($\ell_{50}$), (ii) RALE, and (iii) the RSR. Although the 3 indicators differ in the way deaths and births at different ages are accounted for (see Table 1), we find that, regardless of the selected metric, the contemporary United States consistently performs worse than its peer nations. If mortality and fertility conditions observed in 2019 remained stable, 21 in 1000 newborn girls would die before age 50 years in the other wealthy nations, while in the United States it would be more than twice as many, at 44 in 1000 ($\ell_{50}$). Under 2019 conditions, a girl alive at age 15 years would, on average, live 2.9 fewer months before age 50 years in the United States compared with the other wealthy nations (RALE). Finally, 15 of 1000 potential children would not be born under 2019 conditions in the United States because of premature mortality among potential mothers (RSR). In the other wealthy nations, it would only be about half as many: 8 out of 1000 children. Taken together, the 3 indicators reviewed here paint a comprehensive picture of the negative consequences of mortality before the end of the reproductive period for population-level birth counts in the United States. Whereas the counterfactual population projection approach is most effectively applied with some time lag, the 3 life table indicators are period-based and react instantaneously to changes in the mortality environment. Thus, these indicators can be used to track and communicate year-to-year progress on the US mortality–fertility nexus. We recommend that the choice of the most suitable indicator is based on the metric of interest (probability, years of life, births) as well as the age range accounted for by the different indicators.

Our findings complement existing research that suggests the high burden of disease in the United States may prohibit individuals from starting and raising a family (28). Widening socioeconomic inequalities combined with a limited social safety net, the absence of universal healthcare coverage, and the lax regulation of health threats and threats to life—such as opioids, firearms, environmental pollutants, and unhealthy foods—have played an important role in the emergence of the missing Americans (1), including missing births. In 2019, the causes of death that afflicted women of reproductive age the most in the United States spanned accidents (including accidental drug poisonings), malignant neoplasms, diseases of the heart, suicide, and homicide (29, 30). Moreover, reproductive-aged women in the United States saw increases in a broad group of causes of death over the period 1999–2019, including accidents, suicide, chronic liver disease/cirrhosis, diabetes mellitus, septicemia, and nephritis/nephrotic syndrome/nephrosis (30). Mortality related to pregnancy/childbirth/the puerperium grew by more than 180% across the same period, further pointing to adverse consequences of restricted abortion access and/or access to high-quality maternal care (30, 31). Therefore, following Bor et al., we contend that preventing future missing Americans, including missing births, “will require efforts to address fundamental causes of poor population health in the United States that existed even before the [COVID-19] pandemic began” (1).

There are 2 limitations to the methods suggested in this article. First, we assume that the age-specific fertility rates observed among the surviving individuals in the United States would have applied to the deceased individuals as well, and that all unrealized live births estimated in this way should be counted as missing. However, this assumption does not account for heterogeneity in fertility outcomes, such as health-related fertility differences between surviving and dying individuals (28). Moreover, related to a broader limitation of macro-level fertility research, we are not able to capture the intentionality of births. Compared with other countries in Europe and Northern America, the United States has a high rate of unintended pregnancies (32). This is especially true for groups that have been historically marginalized (33) and that constitute a disproportionate share of the missing Americans. Whether unintended births should be included in a measure of missing births is up for debate. We encourage continued research and dialogue on the best ways to estimate missing births, and missing Americans more generally.

Second, against a history of misinterpretation of demographic period indicators (34), sometimes deliberate, we caution that the 3 life table indicators included here ($\ell_{50}$, RALE, and RSR) only describe the experience of actual birth cohorts if 2 assumptions hold: (i) that all demographic rates remain stable and (ii) that there is no migration. It is rare that these assumptions hold. While there have been various attempts to incorporate migration into demographic indicators of fertility and/or mortality, such as the total fertility rate (35) or the net reproduction rate (36), the assumption of stable demographic rates is almost always violated (37). The indicators suggested here should not be seen as forecasting instruments, but instead as helpful tools to summarize the mortality and fertility conditions in a given year, net of the underlying population age structure (37).

Despite these limitations, the strength of our approach lies in extending the concept of “missing Americans,” as introduced by Bor et al., and in emphasizing the relevance of the mortality–fertility nexus even in high-income countries where mortality before the end of the reproductive period is generally considered low. Going forward, the indicators and methods reviewed in this article could be valuable in monitoring progress toward removing barriers that prevent people from starting and raising a family on their own terms. This includes progress in addressing reproductive inequalities experienced by groups with historically higher rates of early mortality, such as those from lower socioeconomic backgrounds and some historically marginalized racial or ethnic groups in the United States, as these groups may face the greatest burden of missing births. Whether or not this means that preventing premature mortality will lead to larger birth cohorts—as those involved in depopulation discourses may hope or racist ideologues of “hyperfertility” (38) may fear—should not determine if the physical integrity and reproductive autonomy of those currently alive will be guaranteed. An unconditional focus on the health of those alive today will allow individuals to participate actively in society, embedded in kinship and social networks that are not disrupted by avoidable morbidity or mortality.

## Supplementary Material

pgae058_Supplementary_Data

## Data Availability

Data are publicly available, under a Creative Commons license CC BY 3.0 IGO, from the United Nations World Population Prospects (UNWPP) 2022: https://population.un.org/wpp/. For ease of replication, all R scripts and project data, including the data underlying all figures, are available at our Open Science Framework (OSF) repository: https://doi.org/10.17605/osf.io/z5djb.

## Footnotes

1 In line with the two-sex structure of the data and methodological approaches reviewed and used in this article, we use the terms “girl,” “woman,” and “female” interchangeably to refer to individuals capable of pregnancy. We recognize that our data and methods (i) do not capture all individuals self-identifying with 1 or more of these terms at some or all time points; (ii) capture some individuals not self-identifying with 1 or more of these terms at some or all time points; and (iii) capture some individuals not capable of pregnancy, even when self-identifying with 1 or more of these terms at some or all time points (3, 4).

2 In the literature on missing females, the concepts “missing female births” and “females missing at birth” are used to refer to never-born female children due to sex-selective abortion (5). Thus, “missing females” is a broad concept that comprises the postnatal pathway of female excess mortality and the prenatal pathway of sex-selective abortion. We borrow the term “missing births” in this article to only highlight the indirect consequences of high (male and female) excess mortality in the United States for birth counts.

3 While all approaches discussed in this article can be applied with arbitrary year cutoffs, e.g. July to June, we always refer to the calendar year (January to December).

4 The reproductive period varies between individuals of the same sex and between individuals of different sexes. For females, menopause limits the reproductive period, though this is changing with the widening availability of assisted reproductive technology. Defining the length of the reproductive period is also dependent on data availability, as data providers vary in the lower and upper age limits for which they report births. Still, among both females and males, most births occur during ages 15 to 49 years, which is the most common definition of the reproductive period in demographic research (13).

## Previous presentation

Previous versions of this manuscript were presented at the 6th Human Mortality Database Symposium in June 2022 in Paris, France; at the British Society for Population Studies annual conference in September 2022 in Winchester, United Kingdom; and at the Population Association of America annual meeting in April 2023 in New Orleans, Louisiana.

## Preprint

An earlier version of this manuscript was posted as a preprint on SocArXiv: https://doi.org/10.31235/osf.io/fdj6y.
