# Supplementary material for: The impact of early death on birth counts in the United States, 1950 to 2019
Source: PNAS Nexus. 2024 Jun 7;3(6):pgae058. doi: 10.1093/pnasnexus/pgae058 (PMC11157966; doi:10.1093/pnasnexus/pgae058)
Supplement: pgae058_Supplementary_Data [file pgae058_supplementary_data.docx]

**Supplementary Information**

for

The impact of early death on birth counts
in the United States, 1950–2019

Antonino Polizzi* and Andrea M. Tilstra

* To whom correspondence should be addressed: antonino.polizzi@nuffield.ox.ac.uk

**This file includes:**

- Supplementary Information Text
- Figures S1 to S9

**Supplementary Information Text**

**Methods.** In step I of our counterfactual projection of live births, we compare annual birth counts in a baseline (with US mortality) and a counterfactual (with peer mortality) projection scenario to determine the total annual number of children that *were not* (missing births) or *were only* (additional births) born because the United States did not experience the mortality conditions of other wealthy nations beginning in 1950. In step II of our counterfactual projection of live births, we disaggregate the missing births into children not born because their potential mothers (second generation) or (great-)grandmothers (third and higher generations) died under US mortality conditions but would have survived under peer mortality conditions. Similarly, we disaggregate the additional births into children born only because their mothers (second generation) or (great-)grandmothers (third and higher generations) survived under US mortality conditions but would have died under peer mortality conditions.

To disaggregate missing and additional births by generation, we developed a stepwise counterfactual population projection approach. First, we survive the observed US population in 1950 to the end of the projection period using baseline and counterfactual mortality rates. In both the baseline and the counterfactual scenario, we allow for in-migration and record the total number of children, but not (great-)grandchildren, born. We correct the annual birth counts obtained in this way for out-migration of potential parents. Second, the annual birth counts after correction for out-migration are separated into two components: (a) births that occurred in both the baseline and the counterfactual scenario; (b) births that occurred only in the baseline (additional births) or the counterfactual (missing births) scenario. Third, births that occurred in both the baseline and the counterfactual scenario are themselves survived forward under baseline and counterfactual mortality conditions. Again, the children of these children, but not their (great-)grandchildren, are recorded and separated into two components: (a) births that occurred in both the baseline and the counterfactual scenario; (b) births that occurred only in the baseline (additional births) or the counterfactual (missing births) scenario. Step three is repeated until the end of the projection period is reached. Fourth, the additional and missing births recorded for each year and projection step are summed into separate annual totals of additional and missing births. These are the additional and missing births in the second generation, i.e. children that were only born because their mothers survived under US mortality conditions but would have died under peer mortality conditions, and children that were not born because their potential mothers died under US mortality conditions but would have survived under peer mortality conditions. Finally, we survive the additional births in the second generation forward under baseline mortality conditions and survive the missing births in the second generation forward under counterfactual mortality conditions. This time, we record all descendants of these children. These are the additional and missing births in the third and higher generations, i.e. children that were only born because their (great-)grandmothers survived under US mortality conditions but would have died under peer mortality conditions, and children that were not born because their potential (great-)grandmothers died under US mortality conditions but would have survived under peer mortality conditions. The sum of all additional and missing births in each year (irrespective of their generation) corresponds to the annual difference in live births between a standard baseline and counterfactual population projection scenario from step I.


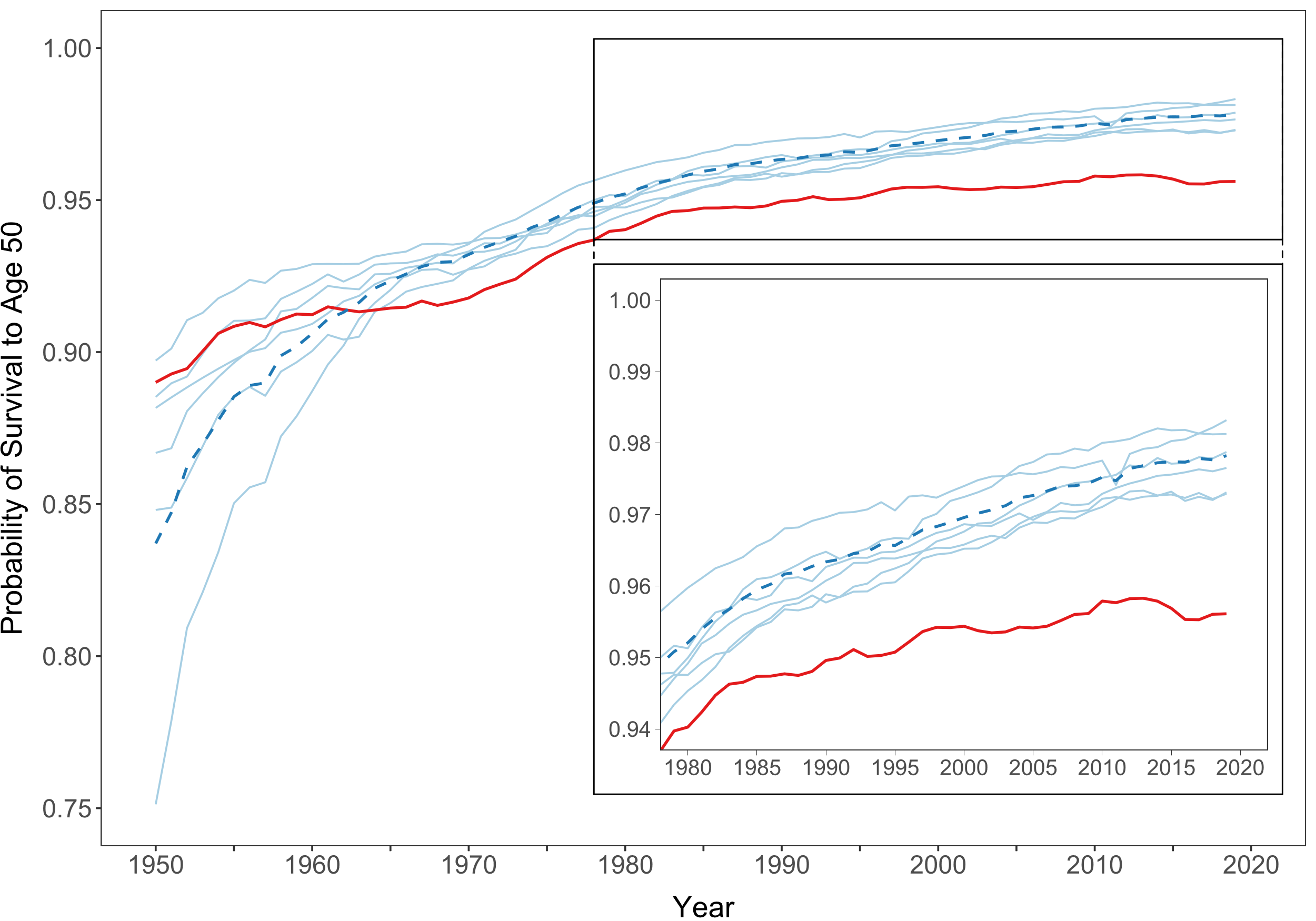


**Fig. S1.** Female probability of survival to age 50 ($\mathcal{l}_{50}$) in the United States and the other Group of Seven (G7) countries, 1950–2019. *Notes*: Thick solid line = United States; thick dashed line = population-weighted average of the other G7 countries; thin solid lines = country-specific trends for each of the other G7 countries. Inset zooms in on period 1980–2019 for better readability. *Source*: Authors’ calculations based on data from United Nations World Population Prospects 2022.


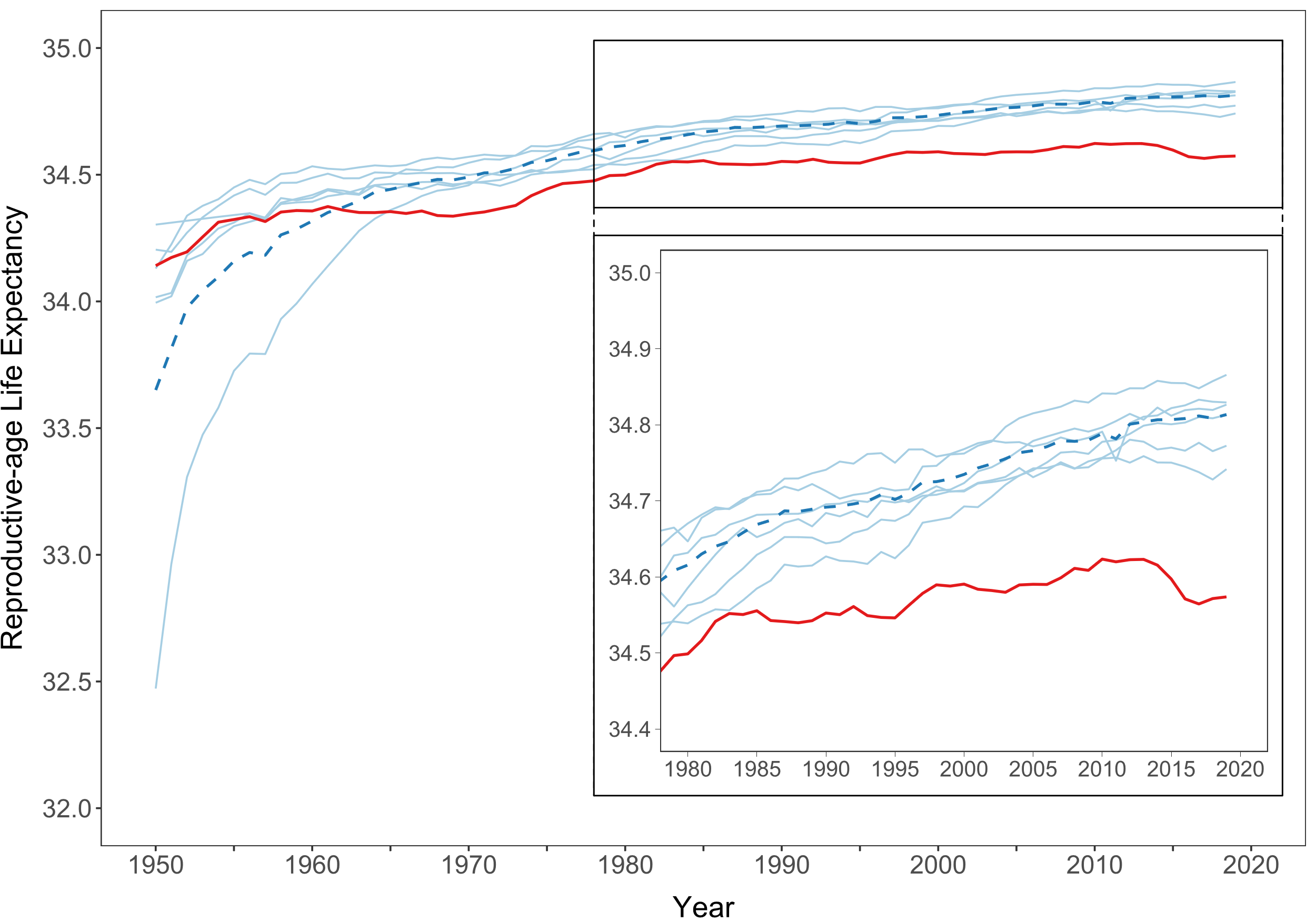


**Fig. S2.** Female reproductive-age life expectancy (RALE) in the United States and the other Group of Seven (G7) countries, 1950–2019. *Notes*: Thick solid line = United States; thick dashed line = population-weighted average of the other G7 countries; thin solid lines = country-specific trends for each of the other G7 countries. Inset zooms in on period 1980–2019 for better readability. *Source*: Authors’ calculations based on data from United Nations World Population Prospects 2022.


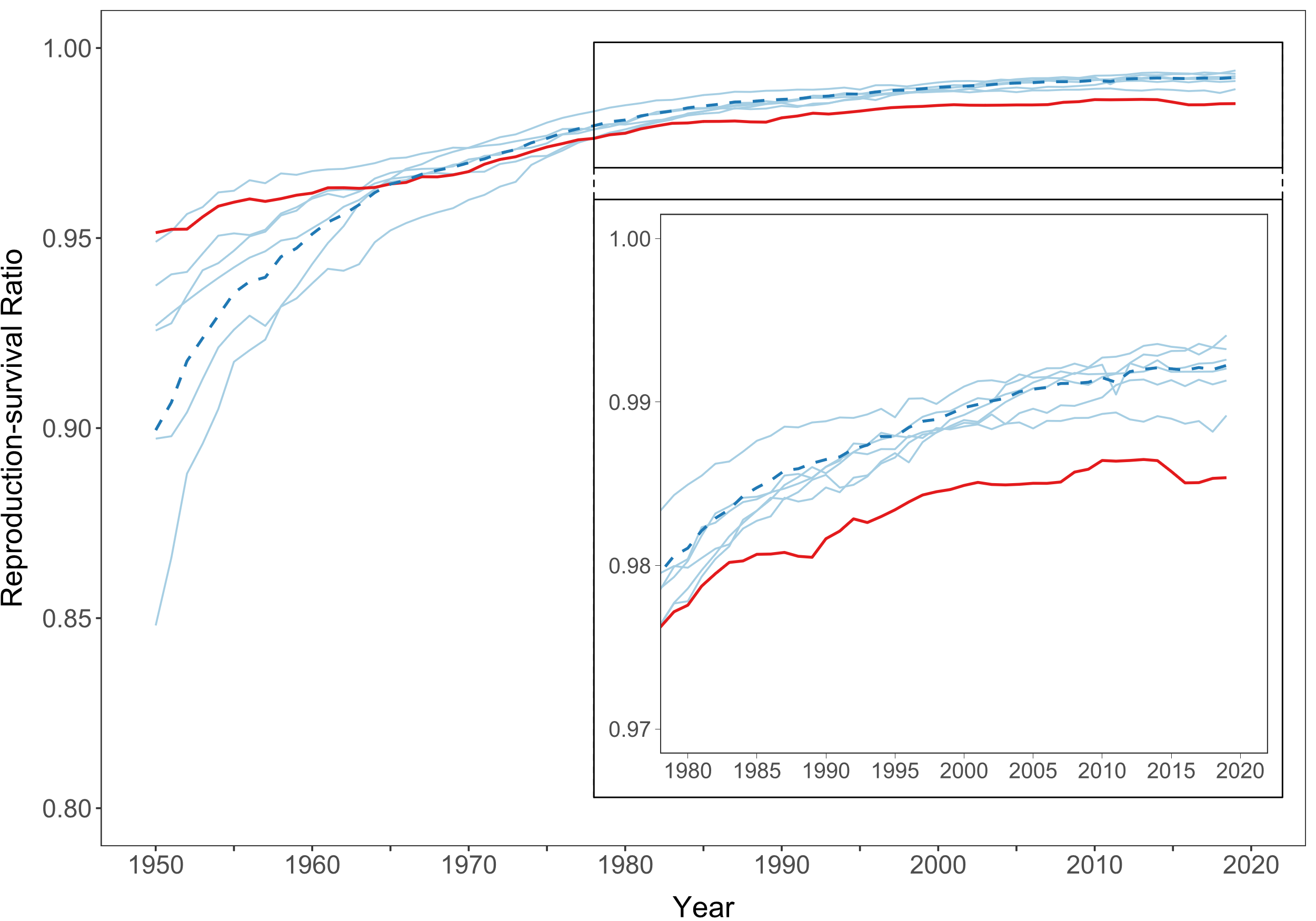


**Fig. S3.** Female reproduction–survival ratio (RSR) in the United States and the other Group of Seven (G7) countries, 1950–2019. *Notes*: Thick solid line = United States; thick dashed line = population-weighted average of the other G7 countries; thin solid lines = country-specific trends for each of the other G7 countries. Inset zooms in on period 1980–2019 for better readability. *Source*: Authors’ calculations based on data from United Nations World Population Prospects 2022.

**
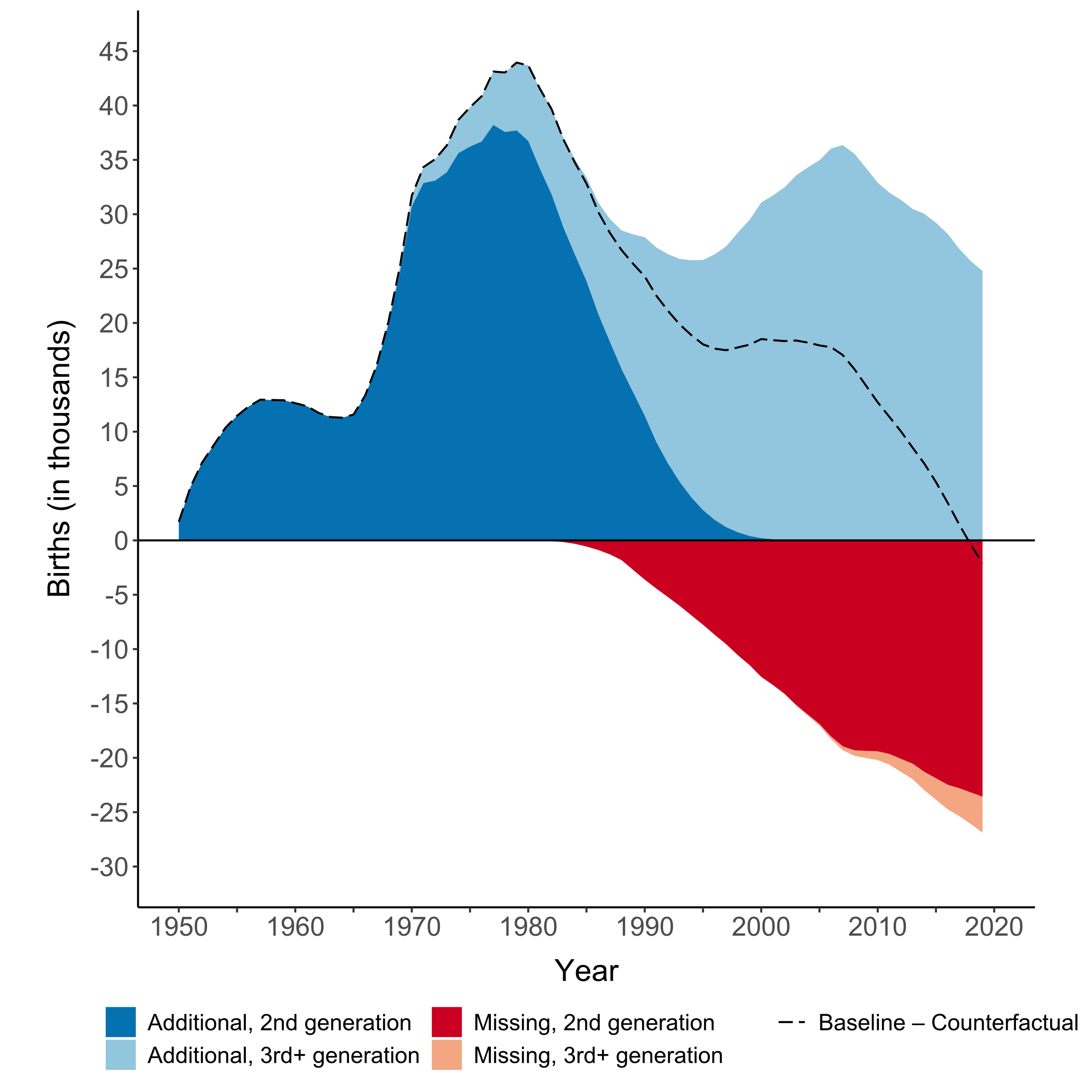
**

**Fig. S4.** Additional births and missing births in the United States, 1950–2019. *Notes*: Areas = children that were only (pointing upward) or were not (pointing downward) born in the United States each year because the country did not experience the mortality conditions of the other Group of Seven countries beginning in 1950. *Source*: Authors’ calculations based on data from United Nations World Population Prospects 2022.


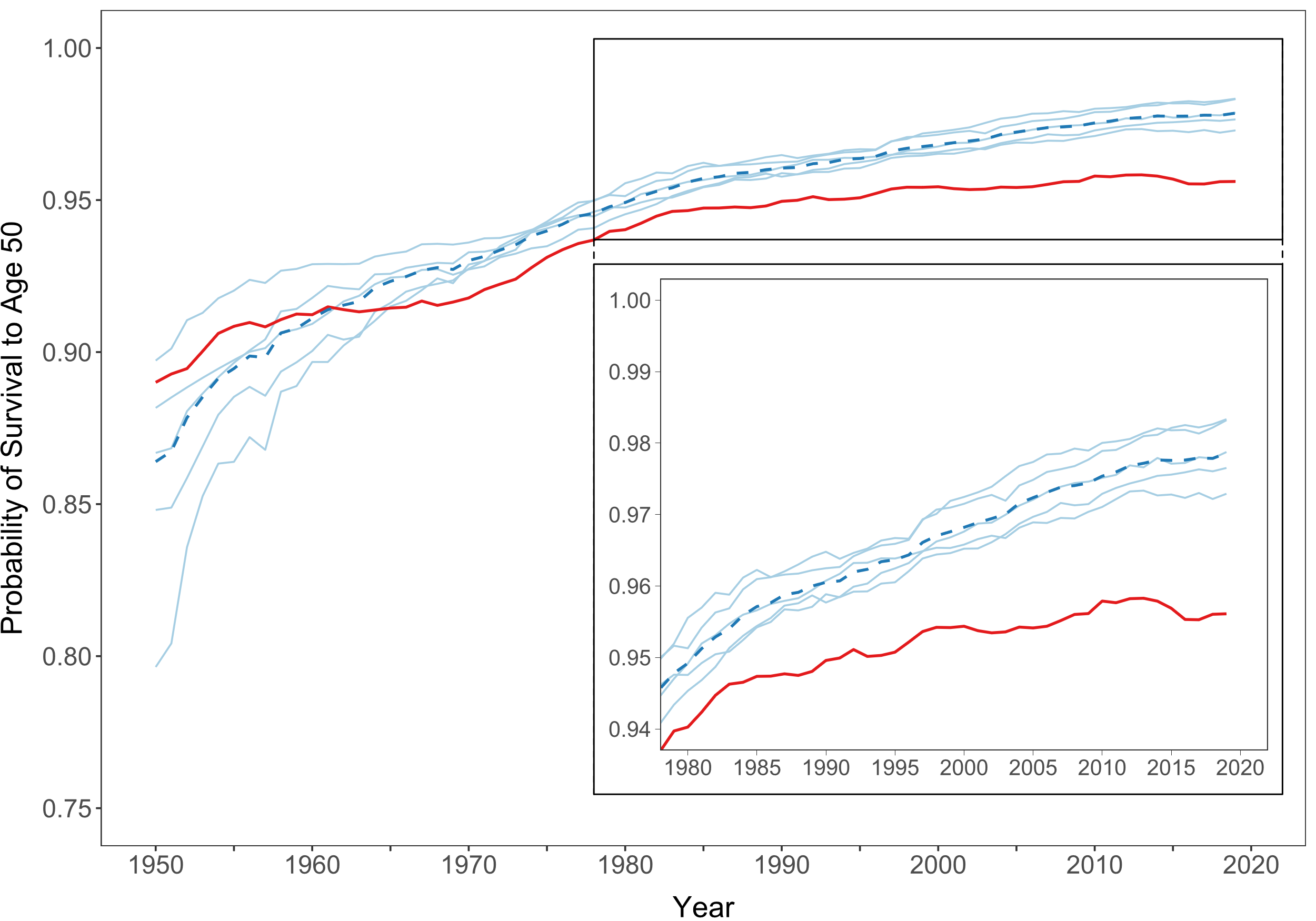


**Fig. S5.** Female probability of survival to age 50 ($\mathcal{l}_{50}$) in the United States and the five largest Western European countries, 1950–2019. *Notes*: Thick solid line = United States; thick dashed line = population-weighted average of the Western European countries; thin solid lines = country-specific trends for each of the Western European countries. Inset zooms in on period 1980–2019 for better readability. *Source*: Authors’ calculations based on data from United Nations World Population Prospects 2022.


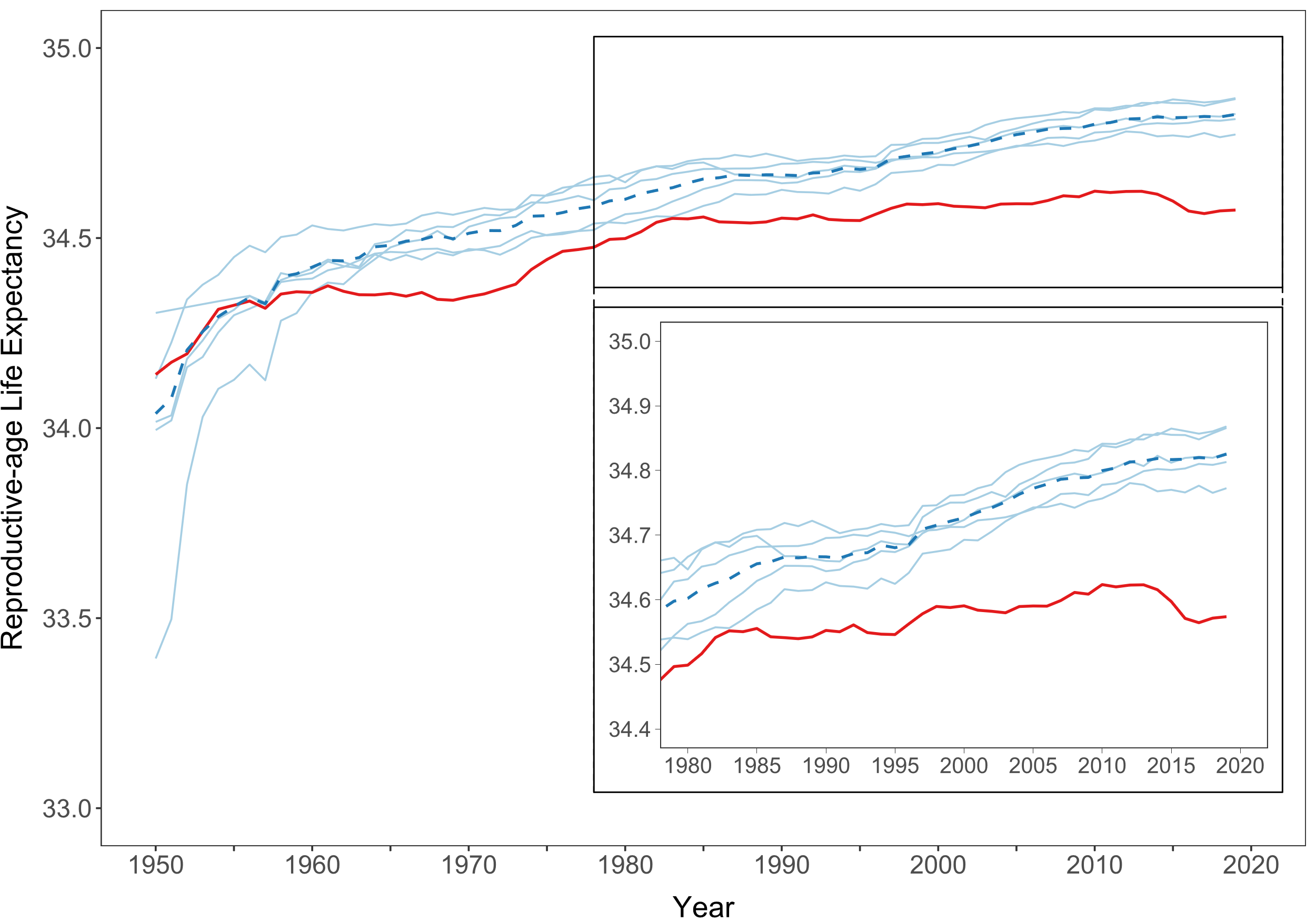


**Fig. S6.** Female reproductive-age life expectancy (RALE) in the United States and the five largest Western European countries, 1950–2019. *Notes*: Thick solid line = United States; thick dashed line = population-weighted average of the Western European countries; thin solid lines = country-specific trends for each of the Western European countries. Inset zooms in on period 1980–2019 for better readability. *Source*: Authors’ calculations based on data from United Nations World Population Prospects 2022.


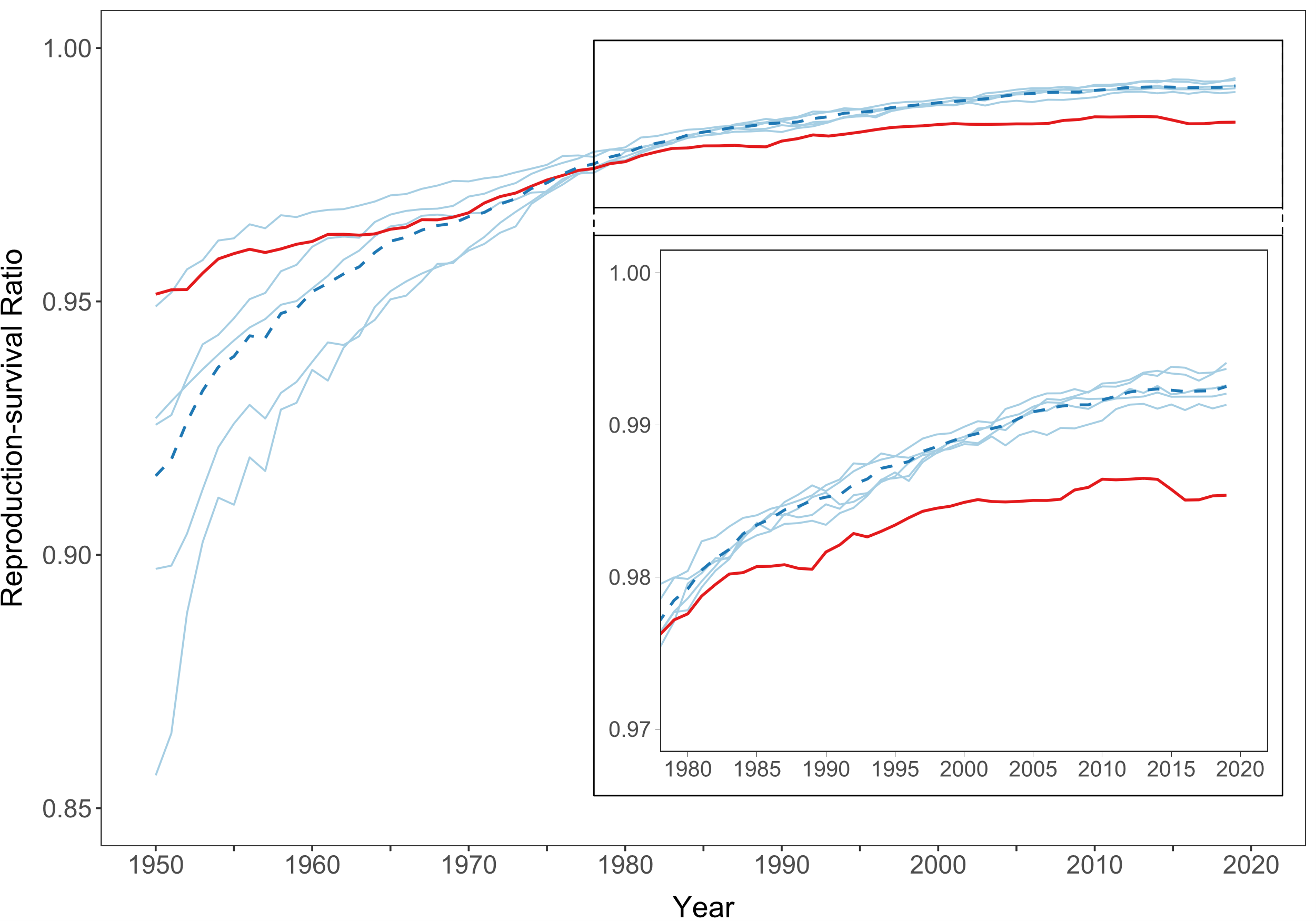


**Fig. S7.** Female reproduction–survival ratio (RSR) in the United States and the five largest Western European countries, 1950–2019. *Notes*: Thick solid line = United States; thick dashed line = population-weighted average of the Western European countries; thin solid lines = country-specific trends for each of the Western European countries. Inset zooms in on period 1980–2019 for better readability. *Source*: Authors’ calculations based on data from United Nations World Population Prospects 2022.

**
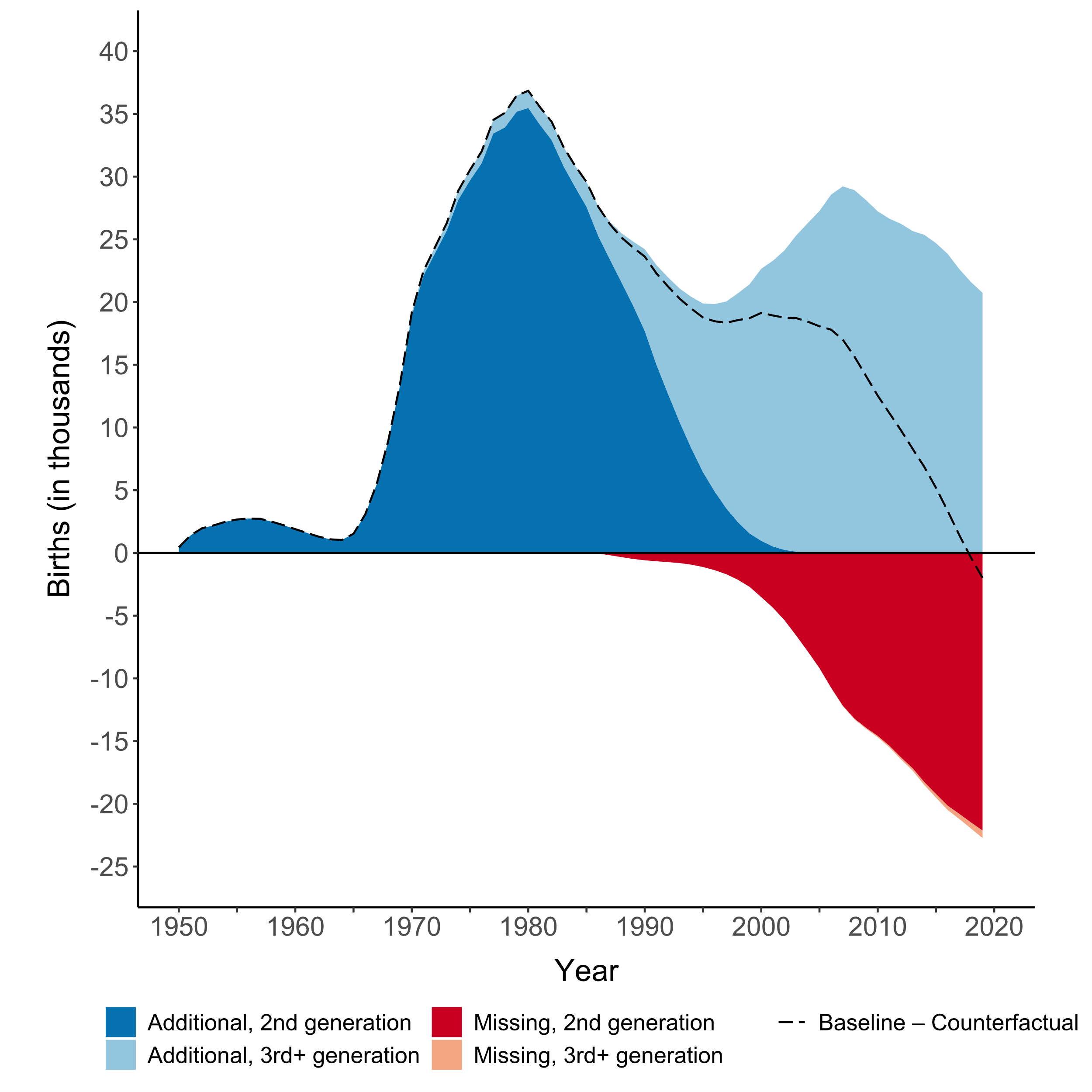
**

**Fig. S8.** Additional births and missing births in the United States, 1950–2019. *Notes*: Areas = children that were only (pointing upward) or were not (pointing downward) born in the United States each year because the country did not experience the mortality conditions of the five largest Western European countries beginning in 1950. *Source*: Authors’ calculations based on data from United Nations World Population Prospects 2022.

**
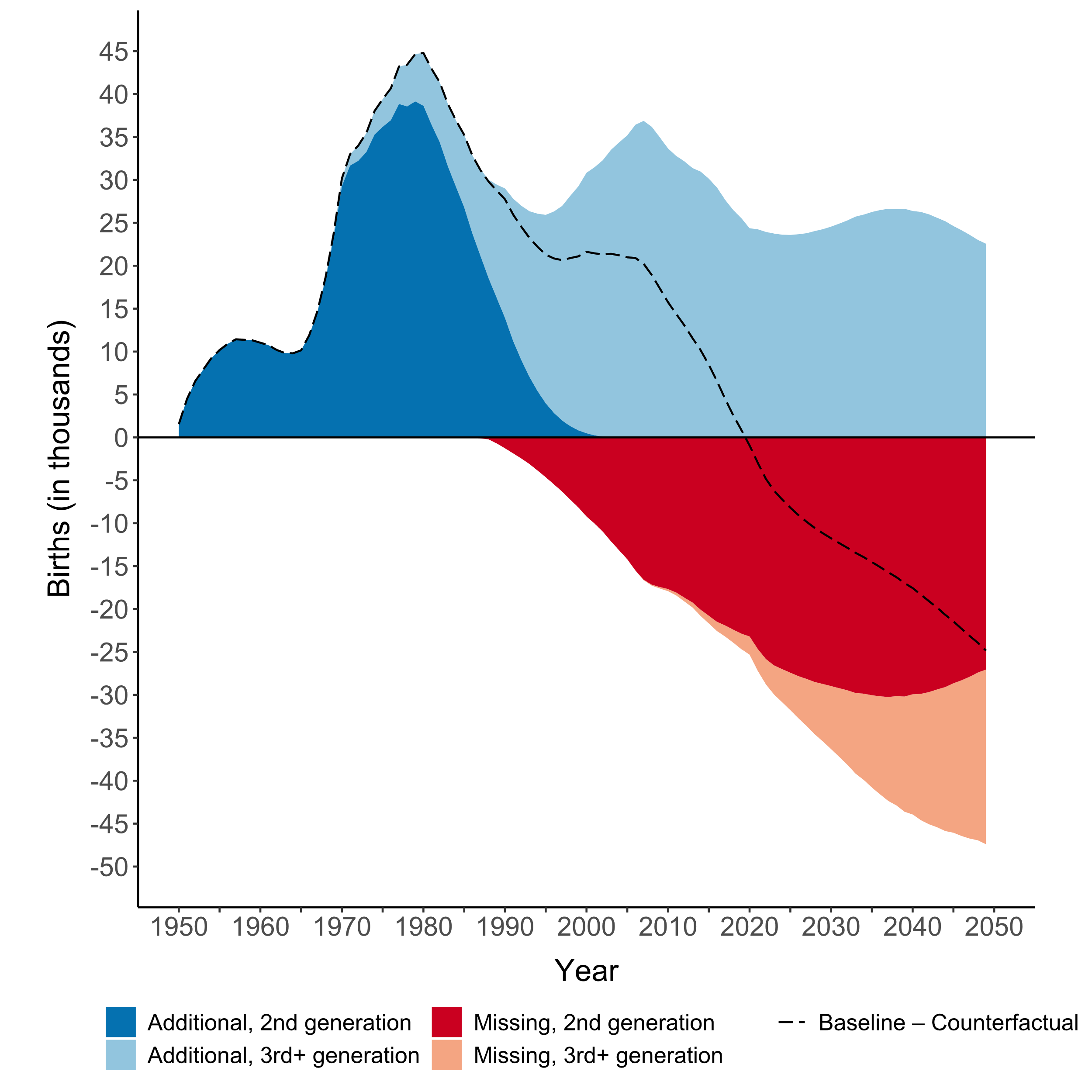
**

**Fig. S9.** Additional births and missing births in the United States, 1950–2049. *Notes*: Areas = children that were only (pointing upward) or were not (pointing downward) born in the United States each year because the country did not experience the mortality conditions of 21 other wealthy nations beginning in 1950. *Source*: Authors’ calculations based on data from United Nations World Population Prospects 2022.
